# Prophylactic G-CSF in patients with early-stage breast cancer: a health economic review

**DOI:** 10.1038/sj.bjc.6605271

**Published:** 2009-09-15

**Authors:** P Trueman

**Affiliations:** 1 York Health Economics Consortium, University of York, York YO10 5NH, UK

**Keywords:** febrile neutropenia, direct costs, indirect costs, prophylaxis, granulocyte colony-stimulating factors (G-CSF)

## Abstract

Although the use of prophylactic granulocyte colony-stimulating factor (G-CSF) in conjunction with myelosuppressive chemotherapy is supported by clinical research evidence and advocated by international clinical guidelines when the consequent risk of febrile neutropenia exceeds 20%, there remains doubt as to the cost-effectiveness of the practice. There are limited economic data, and the data that are available are not necessarily applicable to the management of breast cancer in a European setting. Much of the available evidence on G-CSF in the management of febrile neutropenia is partial, focusing primarily on direct costs to the health service – that is, those related to hospitalisation and drug treatment. A full assessment of the cost effectiveness of G-CSF prophylaxis needs to take account of both costs and outcomes, including mortality, quality of life and patient functioning. As febrile neutropenia has been shown to affect productivity, consideration should also be given to quantifying the indirect costs of neutropenia.

Although the rate of breast cancer has increased in recent decades, patient survival has improved largely as a result of effective chemotherapy regimens (Peto *et al*, 2007), in particular the widespread use of taxanes, such as docetaxel (Peto *et al*, 2007). However, many of the chemotherapeutic regimens that improve patient survival are associated with myelosuppression, which can lead to the development of adverse events, notably neutropenia (Kuderer *et al*, 2006). Neutropenia typically results in fever-like symptoms (febrile neutropenia, FN) and increases patients’ susceptibility to the development of severe infections (Eldar-Lissai *et al*, 2008), which can be life-threatening (Aapro *et al*, 2006). Historically, neutropenia has been managed through chemotherapy dose reductions, designed to reduce the level of myelosuppression. However, such dose reductions can also reduce the clinical effectiveness of chemotherapy and may have a negative impact on patient outcomes.

Use of granulocyte colony-stimulating factors (G-CSFs) is increasingly being recognised as a means of managing FN without resorting to chemotherapy dose reductions (Aapro *et al*, 2006). Evidence for the efficacy of this practice is presented elsewhere in this supplement (Kelly and Wheatley, 2009), and its use in the primary prophylaxis of FN has been endorsed by best practice guidelines. The American Society of Clinical Oncology (ASCO) recommends primary prophylaxis with G-CSF for patients who are at elevated risk of FN, as judged by a combination of factors, including patient age, medical history, disease characteristics and toxicity of the chemotherapy regimen (Smith *et al*, 2006). Although ASCO earlier recommended the use of G-CSF when the risk of FN was greater than 40% (Ozer *et al*, 2000), the latest guidelines use the lower threshold of 20%, in acknowledgement of more recent clinical evidence. Similar recommendations, also based on the 20% threshold, have been issued by both the European Organization for Research and Treatment of Cancer (EORTC) (Aapro *et al*, 2006) and the National Comprehensive Cancer Network (NCCN) (Crawford *et al*, 2009). EORTC also advocates selective use of G-CSF where the risk of FN is estimated to be 10–20%. Guidance on the use of secondary prophylaxis for patients who have previously experienced a neutropenic event is included in the guidelines (Smith *et al*, 2006; Crawford *et al*, 2009).

However, there are concerns about the financial implications of the widespread use of G-CSF in early-stage breast cancers (Ozer *et al*, 2000; Smith *et al*, 2006). ASCO's decision to reduce the FN-risk threshold for primary prophylaxis from 40 to 20%, as well as the endorsements by EORTC and NCCN, has increased the eligible population for G-CSF and the financial costs of using it in accordance with the guidelines. Commentators have questioned whether this extra cost is justified (Adams *et al*, 2006).

This paper attempts to summarise some of the economic implications of the more widespread adoption of G-CSF and considers whether the recommendations presented in the clinical guidelines might be considered cost effective.

A discussion of the costs of using prophylactic antibiotics (as discussed by Cullen and Baijal (2009) in this supplement), either in combination with or in place of G-CSF, is not included. It is sufficient to say that the direct costs associated with antibiotics are largely negligible in the context of the costs of treating breast cancer.

## The costs of FN: direct and indirect

FN is recognised as a costly side effect of chemotherapy for breast cancer. It is associated with direct costs (those incurred by the health-care payer) and indirect costs (those incurred by the patient and caregivers). Table 1 provides a breakdown of some of the costs that should be considered when trying to determine the cost-effectiveness of a preventative approach to FN management. In addition to the direct and indirect costs, consideration also needs to be given to the intangible costs incurred by patients.

Estimates of the direct costs of managing FN vary substantially. They depend on various factors, including the care setting, the particular cancer and the severity of the episode. Studies from the United States tend to report higher costs than do those from Europe.

Bennett and Calhoun (2007) have reviewed both the direct and indirect costs of FN in North America, based on the type of cancer being treated, and on whether FN was managed principally on an outpatient or inpatient basis, in 71 patients recruited from 10 community oncology centres. Among those with breast cancer, FN was associated with a direct cost of $1094 (∼£730) per episode treated in outpatient settings, and $10 354 (∼£6950) per episode treated in inpatient settings. For those treated as inpatients, FN-related hospitalisations accounted for over 75% of the costs, whereas medications accounted for the majority of the costs in outpatients. Indirect costs for patients with breast cancer who developed FN were estimated to be $1530 (∼£1030) for outpatients and $2832 (∼£1900) for inpatients, and were attributed to work loss and caregiver time.

In a retrospective study from Spain, the estimated cost of FN was €3519 (∼£2360) per episode (Mayordomo *et al*, 2006). This is broadly in line with estimates from the United Kingdom, where the reference cost (essentially the charge to the payer) for an admission with FN is approximately £3300–£4300 and a recent review conducted by the National Institute for Health and Clinical Excellence (NICE) estimated a cost of about £3330 per FN episode (NICE, 2008).

## Cost of prophylactic G-CSF

### US perspective

Analysis of more than 24 000 patients hospitalised with FN in the United States, conducted as part of an economic evaluation of G-CSF (Eldar-Lissai *et al*, 2008), indicated that the mean length of stay was 9 days for patients who survived FN and 15 days for those who died as a result of it. The mean cost of FN management per day ranged from $1984 (∼£1330) to $3139 (∼£2100). On the basis of research published earlier, the authors assumed that the use of prophylactic G-CSF would reduce the length of hospital stay by 20%, thus saving several thousand dollars per patient.

The authors acknowledge that their study has a number of limitations, including the use of evidence drawn from a number of different trials, and the application of data to a hypothetical patient population. From a European perspective, the most notable limitation is the higher cost of inpatient FN management in the United States compared with Europe. Evidence from European studies of prophylactic G-CSF (used in combination with antibiotics) suggests that the economic case may be less convincing in European settings, because of the lower cost of managing FN episodes. However, as stated earlier, the cost of managing FN has been found to differ across malignancies, and therefore the relevance of this evidence to breast cancer is uncertain.

### UK perspective

In the United Kingdom, the cost of G-CSF prophylaxis during breast cancer chemotherapy has been estimated at £3100–£5900 per patient, for six cycles (All Wales Medicines Strategy Group, 2008).

Thus, on the basis of direct costs alone, prophylaxis may not compare favourably with the cost of managing an episode of FN as discussed above (e.g., £3330, according to the NICE analysis). However, this crude financial calculation takes no account of the indirect costs of FN, as well as the benefits of prophylactic treatment in terms of avoiding neutropenic episodes and the resultant impact on the individual patient's mortality and quality of life. A full economic evaluation of prophylactic G-CSF should capture both the direct costs (acquisition costs, treatment costs and impact on other health-care resources) and consider the indirect costs (e.g., lost productivity, patient travel time and impact on patient outcomes). A broad perspective that takes account of the full range of costs associated with FN would likely lead to a more favourable conclusion for primary prophylaxis.

## Conclusion

Best practice guidelines from both North America and Europe recommend selective use of G-CSF for primary prophylaxis of FN. There is consensus across the guidelines, which recommend G-CSF for individuals whose risk of FN exceeds 20%.

There is only limited economic evidence for the cost effectiveness of implementing this recommendation, and a great deal of variation between findings from studies in the USA and Europe because of differences in the estimated cost of managing an episode of FN. Further research evidence is needed to allow accurate estimation of the costs of managing FN specifically in patients with breast cancer, in Europe, which seeks to capture the direct, indirect and intangible costs associated with FN. Such research will help to determine the most economically advantageous positioning of G-CSF in the management of breast cancer, so that patients receive the best possible care while scarce health-care resources are allocated appropriately.

## Conflict of interest

P Trueman has received consulting fees from Novartis and Wyeth and has equity ownership/stock options with Johnson & Johnson.

## Figures and Tables

**Table 1:** Costs to be considered when trying to determine the cost effectiveness of a preventative approach to FN management

**Direct costs**	**Indirect costs**	**Intangible costs**
Hospitalisations	Productivity losses	Reduced quality of life for sufferers
Medications	Carer time	
Community/primary care follow-up	Travel costs to health-care consultations	
